# Comparative Analysis of Brain Coping Mechanisms in Small Left-Hemisphere Lesions: Incidental vs. Symptomatic Gliomas

**DOI:** 10.3390/brainsci14090887

**Published:** 2024-08-30

**Authors:** Elisa Cargnelutti, Tamara Ius, Marta Maieron, Serena D’Agostini, Miran Skrap, Barbara Tomasino

**Affiliations:** 1 Scientific Institute, Istituto di Ricovero e Cura a Carattere Scientifico E. Medea, Dipartimento/Unità Operativa Pasian di Prato, 33037 Pasian di Prato, Italy; 2 Neurosurgery Unit, Head-Neck and Neurosciences Department, Azienda Ospedaliero Universitaria Friuli Centrale, 33100 Udine, Italy; tamara.ius@asufc.sanita.fvg.it (T.I.); skrap@asufc.sanita.fvg.it (M.S.); 3 Department of Physics, Azienda Ospedaliero Universitaria Friuli Centrale, 33100 Udine, Italy; marta.maieron@asuiud.sanita.fvg.it; 4 Neuroradiology Unit, Department of Radiology, Azienda Ospedaliero Universitaria Friuli Centrale, 33100 Udine, Italy; serena.dagostini@asufc.sanita.fvg.it

**Keywords:** incidentally discovered low-grade glioma (iLGG), symptomatic glioma, language, neuroplasticity, white matter, object-naming network

## Abstract

Background. Incidentally discovered low-grade gliomas (iLGGs) are very rare and little is still known about their associated functional imaging activation patterns, white-matter status, and plasticity potential. Recent studies shed light on several clinical factors responsible for the good clinical status observed in these patients versus those with their symptomatic counterpart (sLGGs), including small volume. Comparisons were typically carried out by comparing iLGGs with the wider and more heterogeneous sLGG group. In this study, we investigated whether iLGGs affect the brain differently from comparably small sLGGs. Method. Starting from a sample of 13 patients with iLGG, in the current comparative cross-sectional study, we identified a group of patients with sLGGs, primarily matched by lesion volume. We looked for potential differences between the two groups in language-related functional and structural parameters (the fMRI network associated with naming and white-matter fascicles). Results. The *t*-test did not show significant differences in the fMRI network, but these emerged when performing masking. No significant differences were observed at the white-matter level. Conclusions. Given that small volumes characterized both groups and that demographic variables were comparable, too, we hypothesized that differences between the two groups could be attributed to alternative lesion-related parameters. We discussed these findings from clinical and neurosurgical perspectives.

## 1. Introduction

Incidentally discovered low-grade gliomas (iLGGs) are an infrequent condition; indeed, although estimation of their incidence is hard to define, it is normally set at around 0.2–0.3% of the general population [1,2] and up to 10% of all glioma diagnoses [2,3]. For this reason, only recently, studies have focused on the investigation of these lesions, with the aim to shed light on their radiological and histological features and associated long-term prognosis. Incidental lesions, as the name suggests, are accidentally detected when performing radiological examinations for reasons independent from the lesion itself [1,2,3,4,5], given that the patients harboring them do not manifest any evident symptoms. Nevertheless, iLGGs, too, exhibit progressive development, with a median 48-month interval from radiological detection to symptomatic manifestation; this supports early surgical intervention even in asymptomatic cases, with maximal resection recommended [4,6].

Besides the better general clinical status and the absence of symptoms, patients with iLGG, as compared with the symptomatic (sLGG) counterpart, were observed to have longer overall and recurrence-free survival following surgery [2,5,7,8], (but see [9] for the bias in this estimation); this factor was attributed to the very high probability of total or gross total tumor resection, which, further, did not cause postoperative permanent deficits normally [2,6,10,11]. A majority of studies focused on the identification of the factors responsible for this more favorable outcome in patients with iLGGs as compared with sLGGs. Specifically, incidental lesions were observed to have smaller lesion volumes [2,7] (see also [11,12,13]), rarer involvement of eloquent areas [2], an expansive (vs. infiltrative) growth pattern [2,5,11,14], and, probably, a specific molecular profile [7,12,15] (but see [2]).

What is noteworthy is that, in these clinical reports, the small sample of iLGGs was compared to the bigger sLGG group, which is typically more heterogeneous, especially in terms of volume and localization. As a consequence, remarkable differences between the two groups were observed in terms of the above-cited lesion-related characteristics. In particular, iLGGs have been hypothesized to represent the early phase of gliomas, which then evolve into a symptomatic condition, by increasing in volume and affecting additional brain tissue (for instance, by progressively invading the white-matter structures) [14,15]. This implies that lesion volume is a key factor in discriminating between the silent and the symptomatic condition. As a consequence, a key question is whether significant changes could be detected when running a comparative cross-sectional analysis, meaning when iLGGs and sLGGs are matched as closely as possible in terms of lesion volume in the first place. Nevertheless, even though, overall, symptomatic lesions are larger than incidental lesions, they can be characterized by small volumes, too.

Another crucial point is that little is still known about the functional imaging activation patterns and white-matter status consequent to iLGG growth and even less is known about their plasticity potential, which has been instead widely reported for LGGs in general [16,17,18,19]. This issue is very relevant as these patients, besides a normal clinical status, are also generally preserved from the cognitive viewpoint [20,21]; this means that, at the stage of diagnosis, the tumor had not harmed the cognitive performance yet (although there is not a convergent agreement on this [22]); in two previous studies, in which we addressed the white-matter status [23] and the fMRI network associated with object naming [24] in patients with a left iLGG, we did not observe substantial differences between them and healthy controls.

In the current study, then, we aimed to perform a more thorough investigation by comparing patients with incidental and symptomatic lesions in the left, language-dominant hemisphere; then, we carried out an exploratory comparative group-balanced cross-sectional investigation, in which we aimed to shed light, in particular, on the role of lesion volume. Our main research question was as follows: at the neurological level, is there any difference between iLGGs and sLGGs when the latter, too, are characterized by the small volumes (and are located in the same macroanatomical areas) of the iLGGs? The investigation addressed language functions; therefore, we explored the potential differences between the two groups in the functional network associated with object naming and in the parameters (i.e., FA and number of streamlines) of the main language-related white-matter fascicles (i.e., the inferior longitudinal fasciculus (ILF), inferior fronto-occipital fasciculus (IFOF), uncinate fasciculus (UF), and arcuate fasciculus (AF)).

## 2. Materials and Methods

### 2.1. Participants

In Cargnelutti et al. [24], we identified, among the whole sample of patients operated on for a glioma since the year 2000, a cohort of 13 patients with iLGG in the left hemisphere and with complete neuroimaging and neuropsychological examinations. No additional patients met these criteria at the time of the current investigation; then, the final sample of patients with iLGG was represented by those same 13 patients in [24]. For the purposes of the current study, we selected 13 patients harboring an sLGG in the left hemisphere in order for them to be matched, as much as possible, with the iLGG group, primarily in terms of lesion volume. To exclude the role of lesion location, we ran the selection in order for the two groups to be also matched in terms of macroanatomical location (i.e., nine frontal, three temporal, and one parietal).

**Figure 1:**
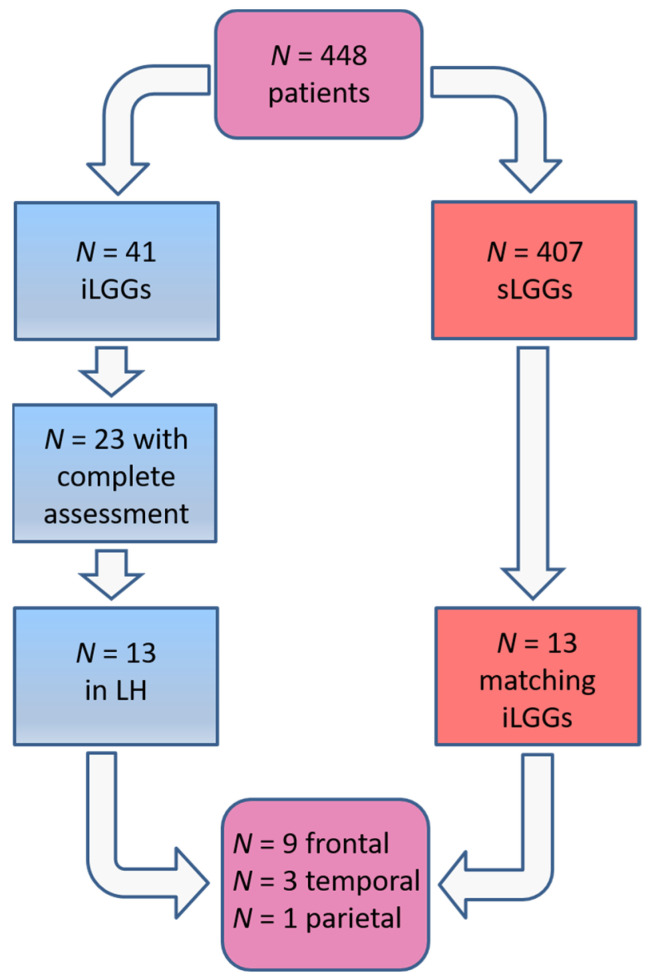
Patients’ selection procedure.

**Figure 2:**
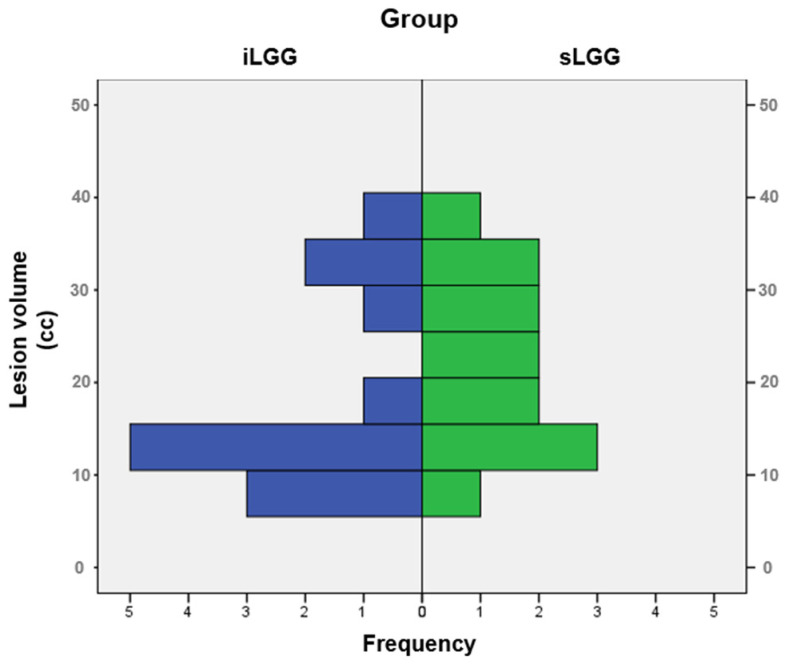
Frequency distribution of lesion volumes in the iLGG and sLGG groups.

The selection procedure of the patients with sLGG, starting from the total amount of 448 patients operated on in the 2000–2024 period (of whom 41 had iLGGs and 407 had sLGGs), is depicted in Figure 1. In detail, we filtered from our database the patients with small lesion volumes in the left hemisphere. The selection was performed at the group level, not following a 1:1 matching. The choice was limited and the final sample represented the best matching, given that no additional patients with sLGG met both the criteria of volume and location comparable with those with iLGG. As it can be seen in Table 1, mean lesion volume did not significantly differ between the two groups (also see Figure 2). Further, we observed that the two groups did not differ significantly in terms of relevant demographic variables (i.e., gender, age, years of education) or handedness [25]. Details are reported in Table 1 (data for the iLGG group can be found in [24]). From a neuropsychological viewpoint, both groups achieved a normal performance in the object-naming test (i.e., a score ≥ 28/30 in the BADA subtest [26]). Concerning the cognitive profile in general, this was mostly preserved in the patients of both groups, with only a few exceptions, and performance did not significantly differ between them (see Supplementary Table S1).

In agreement with the Declaration of Helsinki, all the participants signed an informed consent form to participate in the study, involving both the neuroimaging examination and neuropsychological, normally testing taking place 6–10 days before surgery. The Friuli-Venezia Giulia Unique Regional Ethical Committee (protocol No. 7259, ID 2219) approved the study on the date 27 February 2018.

**Table 2:** Individual sLGG clinical and demographic information.

	Lesion Site	Vol (cm^3^)	Gender	Age	Edu	Hand	Naming (/30)	Symptomatology
p1	Frontal	18	F	73	5	100	29	Speech arrest, R arm myoclonus
p2	Frontal	13	M	28	13	100	30	Epileptic attacks
p3	Temporal	30	M	43	11	100	28	Auditory hallucinations
p4	Temporal	10	F	48	18	100	30	Aphasia, anomia
p5	Parietal	15	F	46	18	100	28	Epileptic attacks, paresthesia (lips)
p6	Frontal	33	F	36	13	−50	30	Epileptic attacks, fatigue, olfactory hallucinations
p7	Frontal	16	M	45	13	100	30	Epileptic attacks, production aphasia, paresthesia (R arm)
p8	Frontal	32	M	40	5	100	30	Epileptic attacks, brain fog
p9	Frontal	36	M	36	8	100	30	Anomia, production aphasia
p10	Frontal	22	M	49	8	100	29	Epileptic attacks, phonological dysarthria
p11	Frontal	23	M	44	13	100	30	Epileptic attacks
p12	Temporal	15	M	42	13	96.67	30	Epileptic attacks, anomia, phonological dysarthria
p13	Frontal	27	F	35	18	100	28	Epileptic attacks

Note. Edu = education (years); Hand = handedness (−100 = perfect left-handedness to 100 = perfect right-handedness), tested by the Edinburgh Inventory [25]. *p* = patient; Vol = lesion volume computed on T2-weighted images. Naming corresponds to the score achieved in the object-naming task (30 items, cut-off score = 28) from the BADA battery [26].

### 2.2. Imaging Data

Structural and functional imaging data were acquired by a 3-T Philips Achieva (Best, Netherlands) whole-body scanner and a SENSE-Head-8 channel head coil; head movements were minimized by a custom-built head restrainer. We acquired high-resolution T2-weighted anatomical MR images (by applying a 3D magnetization-prepared rapid acquisition gradient fast field echo pulse sequence: TR = 2500 ms; TE = 35 ms; FOV = 240 × 240 mm^2^; 190 sagittal slices of 1 mm thickness; flip angle = 90°; voxel size: 1 × 1 × 1 mm^3^) and post-gadolinium T1-weighted anatomical MR images (by applying a T1-weighted 3D magnetization-prepared rapid acquisition gradient fast field echo pulse sequence: TR = 8.2 s; TE = 3.7 s; FOV = 240 × 240 mm^2^; 190 sagittal slices of 1 mm thickness; flip angle = 8°; voxel size: 1 × 1 × 1 mm^3^).

### 2.3. MRI Structural Analysis

Lesion volume was computed by performing tumor segmentation manually across all axial post-gadolinium T1-weighted and all axial T2-weighted slices using the OsiriX software tool (version 9.0) [27]. In order to localize the lesion site, we drew the volumes of interest (VOIs) of the patients’ lesions on their T2 MRI scans using the MRIcroN software, v. 1.0.20190902 (https://www.nitrc.org/projects/mricron). We used the normalization procedure of the “Clinical Toolbox” (https://www.nitrc.org/projects/clinicaltbx/) running on SPM12 (https://www.fil.ion.ucl.ac.uk/spm/) to normalize the VOIs to the Montreal Neurological Institute (MNI) space. We visually inspected the resultant output to exclude errors in the normalization procedure.

The lesion growth pattern was defined by an expert neuroradiologist (S.D.A.), who visually inspected post-contrast T1-weighted and T2-weighted MR images. An expansive pattern was defined for lesions with sharp borders clearly separating the lesion from the surrounding healthy tissue; otherwise (i.e., no sharp borders and with lesion intermingling with the healthy tissue), the growth pattern was defined as infiltrative.

### 2.4. fMRI Data Acquisition

A single-shot gradient echo, echoplanar imaging (EPI) sequence was used for functional image acquisition. EPI volumes (*N* = 54) included 34 contiguous axial slices (RT = 2500 ms; TE = 35 ms; FOV = 230 × 230 mm^2^; matrix: 128 × 128; flip angle = 90°; voxel size: 1.797 × 1.797 × 3 mm^3^); four dummy images allowed the MR scanner to reach the steady state.

#### fMRI Naming Task

During the fMRI session, both patients and controls were instructed to covertly name the pictured objects projected on the screen. The instructions were: “Silently name the picture as accurately and as quickly as possible”. Images were arranged in a block design, alternating four naming blocks and five resting blocks (i.e., cross-fixation); each block lasted 15 s. We used the stimuli from Snodgrass and Vanderwart’s battery [28], whose names were represented by bi- and tri-syllabic words (mean word length in letters = 6.8, *SD* = 2.2; mean length in syllables 2.8, *SD* = 0.89; mean frequency = 1.4, *SD* =1.6).

Stimulus presentation and synchronization with the MR scanner were performed using the Presentation software (Version 9.9, Neurobehavioral Systems Inc., San Francisco Bay Area, CA, USA); stimulus display occurred using the VisuaStim Goggles system (Resonance Technology, Northridge, CA, USA).

### 2.5. Statistical Analyses

We used the IBM SPSS Statistics software (IBM, Armonk, NY, USA), version 21.0, to run the statistical analyses. In particular, the non-parametric Mann–Whitney test was run to investigate between-group differences in numbers of streamlines and FA values for each hemisphere.

### 2.6. fMRI Data Processing

We performed the functional imaging analyses using MATLAB r2018a (The Mathworks Inc., Natick, MA, USA) and SPM12 (Statistical Parametric Mapping software, SPM; Wellcome Department of Imaging Neuroscience, London, UK, http://www.fil.ion.ucl.ac.uk/spm). We excluded dummy images from image processing. We performed image pre-processing using the following steps: spatial realignment to reference volume, segmentation, and normalization to the standard EPI template of the Montreal Neurological Institute. We resampled images to a 2 × 2 × 2 mm^3^ voxel size and spatially smoothed them with a 6-mm FWHM Gaussian kernel.

As we previously did for patients with iLGG [24], the object-naming network for each participant in the sLGG group was achieved in first-level analysis, by modeling the alternating epochs with a simple boxcar reference vector. A general linear model was applied to each voxel for alternating object-naming and baseline conditions; reference waveforms, corresponding to boxcar functions convolved with a hemodynamic response function, modeled the temporal derivatives [29,30]. The realignment procedure used six additional regressors to model the head movement parameters. Linear contrasts between object-naming and baseline conditions defined the design matrix; this procedure provided *t*-statistics for each voxel. A cut-off period of 128 s was used to filter low-frequency signal drifts. *T-*statistics were then transformed into *z*-statistics constituting statistical parametric maps (SPM{*Z*}) of differences between object-naming and baseline conditions.

In second-level random effects analysis, contrast images from each participant were entered into a one-sample *t*-test to create a SPM{*T*} for each contrast at the group level. In detail, we computed the object-naming SPM{*T*} by including the task > baseline contrast from each patient in the sLGG group (as we did for the iLGG group in [24]).

Concerning potential between-group differences, we entered, on SPM12, the first-level task > baseline contrasts separately for each group, and ran the following second-level two-sample *t*-tests: sLGG > iLGG and iLGG > sLGG. In addition, we further explored potential group differences by using a more sophisticated approach, aimed at detecting subtler differences; inclusive masking was devoted to the detection of the voxels significantly activated by both groups, whereas exclusive masking was for the identification of the potential voxels significantly activated in one group but not in the other.

For both analyses, the displayed results were corrected for multiple comparisons at the cluster level (i.e., family-wise error (FWE) correction, *p* < 0.05), with a height threshold of *p* < 0.001, uncorrected, at the voxel level. We used the SPM Anatomy toolbox [31] to define the macroanatomical localization of the functional activation and, when provided, the cytoarchitectonic localization.

### 2.7. DTI Data Acquisition and Analysis

We adopted an axial diffusion-weighted, single-shot, spin-echoplanar imaging sequence covering the whole brain (RT = 8880 ms; TE = 70 ms, bandwidth = 3135 Hz/pixel; matrix size = 128 × 128 voxels; field of view = 240 × 240 mm^2^; flip angle = 90°; slice thickness = 2.1 mm; contiguous axial slices = 57) for diffusion tensor image acquisition. Two *b*-values were used: 0 s/mm^2^ (seven non-diffusion-weighted images) and 1000 s/mm^2^ (64 non-coplanar diffusion-weighted images). Gradient directions were uniformly distributed on a sphere.

We performed tract reconstruction using the DTIStudio software (version 3.0.3, 2010; John Hopkins University, Baltimore, MD, USA). This software conducts deterministic fiber assignment using the continuous tracking (FACT) algorithm. We set the following criteria for reconstruction: maximum turning angle = 70° and FA = 0.15 as a threshold for both start and stop tracking [32]. We visually inspected the tracked fascicles to remove fiber with a potentially anomalous trajectory.

We reconstructed the following fascicles involved in language and passing through or close to the areas affected by the brain lesion: the inferior longitudinal fasciculus (ILF), inferior fronto-occipital fasciculus (IFOF), uncinate fasciculus (UF), and arcuate fasciculus (AF), both the whole fascicle and the long direct segment [33]. The direct AF segment is the classical arcuate pathway connecting Wernicke’s and Broca’s areas directly. We reconstructed the fascicles in both hemispheres by adopting the multi-ROI approach proposed by Catani et al. [33,34]. For the purposes of this study, we recorded the following DTI parameters: the number of streamlines associated with each fascicle and the fractional anisotropy (FA) values.

## 3. Results

### 3.1. Clinical Data

Patients with iLGG and sLGG were characterized by different neurological profiles. In particular, contrary to the patients in the iLGG group who did not manifest any symptoms, patients with sLGG displayed several symptoms and were all characterized by the first clinical manifestation represented by generalized epileptic seizures. In particular, at onset, seven out of thirteen patients (54%) had language-related symptoms (see Table 2 for details).

Interestingly, both groups had a comparable lesion growth pattern, which was expansive for all patients but one in each group (i.e., p3 in iLGG and p9 in sLGG). When addressing potential differences between the two groups regarding the relative involvements of cortical areas and subcortical white matter, we observed that, for both groups, the lesions mainly involved the cortical areas (*M* = 26.53 cc, *SD* = 23.84 for iLGG and *M* = 27.42 cc, *SD* = 16.48 for sLGG vs. white matter: *M* = 3.15 cc, *SD* = 3.99 for iLGG and *M* = 2.02 cc, *SD* = 2.53 for sLGG) and that the two groups did not significantly differ in the amount of involvement of cortical and subcortical structures (respectively, *t* = −0.11, *p* = 0.91; *t* = −0.86, *p* = 0.40).

**Figure 3:**
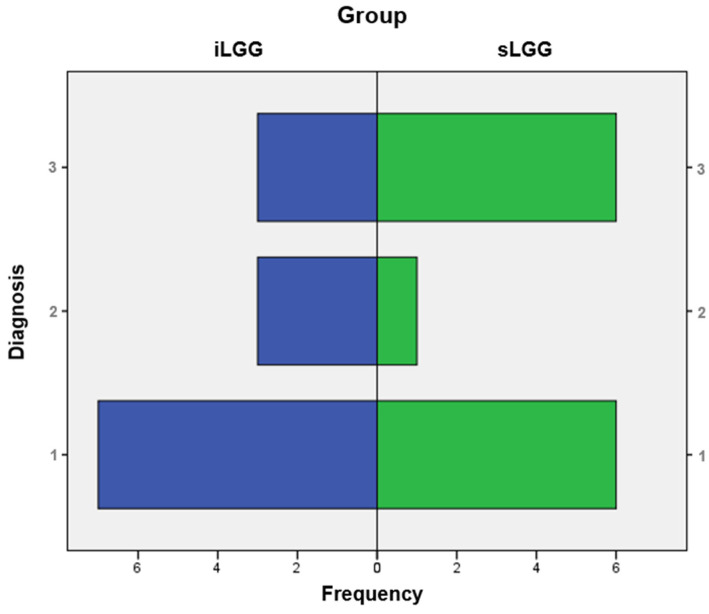
Frequency distribution of molecular diagnoses in the iLGG and sLGG groups. Note. IDH = isocitrate dehydrogenase. For diagnosis, 1: IDH-mutant, 1p/19q codeleted oligodendroglioma; 2: IDH-wildtype diffuse astrocytoma; and 3: IDH-mutant diffuse astrocytoma.

From the molecular viewpoint (see Figure 3), the iLGG group was characterized by 3/13 IDH-mutant, 1p/19q codeleted oligodendrogliomas, 3/13 IDH-wild-type diffuse astrocytomas, and 7/13 IDH-mutant diffuse astrocytomas, which were, respectively, 6/13, 1/13, and 6/13 in the sLGG group. Importantly, the Fisher–Freeman–Halton test showed that this variable was not significantly different between the two groups (value = 1.99, *p* = 0.34).

### 3.2. Functional Naming Network

The functional naming network of the sLGG group is reported in Supplementary Table S2 (for the iLGG group, see [24]) and Supplementary Figure S1A (Supplementary Figure S1B shows the overlap between the networks of the two groups).

**Figure 4:**
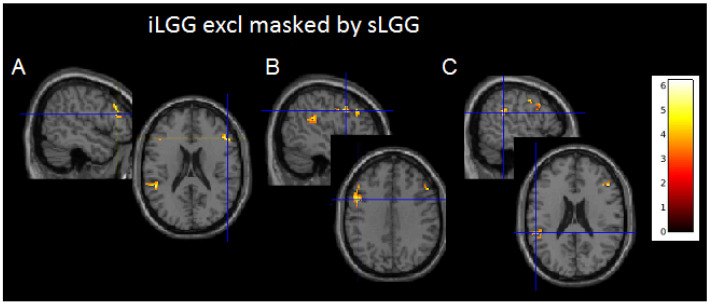
Axial projections of the three functional clusters resulting from exclusive (iLGG by sLGG) masking. In (**A**), left inferior parietal/superior temporal gyri; in (**B**), left inferior/middle frontal gyri; and in (**C**), right inferior/middle frontal gyri. Note. Color bar indicates signal intensity.

Although suprathresholded clusters did not emerge from sLGG > iLGG and iLGG > sLGG comparisons, interesting findings were provided by exclusive masking (see Table 3 and Figure 4 and Supplementary Figure S1C): the iLGG network masked by the sLGG network provided three suprathresholded clusters, two in the affected hemisphere (one in the inferior parietal/superior temporal gyri and the other in the inferior/middle frontal gyri) and one in the right hemisphere (centered to the inferior/middle frontal gyri). In the affected hemisphere, we detected a very low signal in correspondence with the peak activation in the sLGG but not the iLGG group (1.47 vs. 6.83 in the temporo-parietal cluster and 1.47 vs. 5.73 in the frontal cluster). The sLGG network masked by the iLGG network did not provide any suprathresholded clusters.

We also reported the results from inclusive masking, which showed that the two resultant networks (iLGG masked inclusively by sLGG and sLGG masked inclusively by iLGG) were bilateral and highly superimposable, and included primarily the basal-temporal areas, parietal lobe, and inferior frontal gyrus/premotor areas (see Table 4 and Table 5 and Supplementary Figure S1D for details).

### 3.3. DTI Parameters

DTI data were available for all the patients in the iLGG group and for 11 out of 13 patients in the sLGG group. The Mann–Whitney test did not reveal any significant differences for either numbers of streamlines or FA in each hemisphere (Table 6).

## 4. Discussion

Data concerning iLGGs are currently still scarce, given that they represent a very rare condition with an estimated incidence at around 0.2–0.3% [1,2]. However, good clinical and cognitive statuses [20,21] of incidental lesions were recently attributed to several features characterizing them. Given that these lesions probably represent the initial stage in the development of LGGs [14], it derives that their small volume might be a critical factor. Actually, previous studies identified the small volume as a key feature of iLGGs, together with other peculiar lesion-related variables [2,7,11,12,13]. Interestingly, in all these studies, iLGGs were compared to the whole sample of sLGGs, which was typically more heterogeneous under several variables.

With this study, we aimed to increase knowledge about iLGGs with respect to previous studies, including ours, in which we compared these patients with healthy controls. Indeed, in Cargnelutti et al. [24], we addressed the functional network associated with object naming and did not observe remarkable differences between patients with iLGG and healthy controls. The same occurred in relation to their white matter [23]. We attributed this lack of remarkable differences to the fact that iLGGs, probably due to their small volumes especially, had not affected the brain substantially, so they had not yet elicited compensatory plasticity processes.

In the present study, on the contrary, we aimed to compare patients with iLGG to those with sLGG by matching the two groups, as much as possible, by lesion volume. We also controlled for macroanatomical localization in order to reduce sources of variability. Interestingly, although symptomatic lesions are more likely to have larger volumes than incidental lesions [2,15], a first noteworthy result is that even symptomatic lesions may be very small (the smallest in our sample was 10 cc). This suggests that symptom onset could be, at least in part, independent from lesion size.

Further, concerning the other lesion-related parameter, namely the lesion growth pattern, we observed that it was equivalent between the two groups, and specifically that it was expansive for all patients, except for one per group (for whom it was infiltrative). On the contrary, previous studies [2,5,11,12] showed that patients with sLGG more likely harbored infiltrative lesions (vs. the expansive lesions of patients with iLGG) and that this factor contributed to poorer clinical status and surgery success in these patients [2,5,11,12].

To this respect, we can hypothesize that, at the beginning, gliomas grow following an expansive pattern, to become infiltrating only in the second moment. Concerning the hypothesis according to which the lesions become symptomatic when they begin to involve the subcortical white-matter structures [14,15], the mainly expansive growth pattern observed in the sLGGs too, together with the lack of a significant difference concerning the white-matter involvement between iLGGs and sLGGs, suggests that this is not likely to be the case.

The molecular profile was not perfectly matched between the two groups, even though it did not differ significantly. Notably, contrary to previous reports [7,12,15], we observed that the diagnosis of IDH-mutant astrocytoma and oligodendroglioma were equally prevalent conditions in the sLGG group, whereas iLGGs were mainly IDH-mutant astrocytomas. Nevertheless, discussion about the molecular profile is beyond the scope of this paper.

When dealing specifically with language and the related functional and structural parameters, we detected interesting findings. We addressed, specifically, the functional network associated with object naming and the language-related white-matter fascicles (i.e., ILF, IFOF, UF, and AF, both the whole fascicle and the long direct segment). Besides the fact it is tested broadly in our patients, we focused on object naming also because it is a compound task, requiring many areas to be activated, particularly in the left fronto-temporal areas (especially for the related phonological and articulatory processes [35,36,37,38]) that represented the main location of the LGGs in the current investigation. For this reason, object naming is a suitable task to investigate whether a lesion has affected the brain functional systems and possibly prompted compensatory neuroplasticity [16,17].

Regarding the functional imaging viewpoint, surprisingly and contrary to our expectations, we found that the iLGG network included three clusters which were not activated in the sLGG group. The latter, on the other hand, did not activate any additional clusters with respect to the functional map of the iLGGs.

Given that these differences emerged with a more fine-grained analysis, we discuss them with caution. Nevertheless, we reported them as they are prone to an interesting interpretation. Indeed, it was noteworthy that two clusters were located in the left affected hemisphere, and one in the right healthy hemisphere. The fact that these areas were not activated in the sLGG group may indicate a loss of signal in this group or, alternatively, an initial, subtle compensation by the iLGGs, which is confirmed by the activation of a contralesional site in the homolog counterpart of the inferior/middle frontal gyrus.

Taken together, these findings may indicate that the symptomatic lesions, despite being small in size, appeared not to elicit successful compensation before symptom onset, contrary to the incidental lesions, which probably remain silent for a longer period of time due to the development of successful compensation. Interestingly, plasticity seemed to not occur in a hierarchical manner, as previously described [39], but could enroll the contralesional hemisphere in the earlier phases already. The fact that both groups had retained good naming (and general cognitive) skills can suggest that the lack of activation in the sLGG group was marginal at that moment. Indeed, it was only detected with a finer analysis and not with the traditional *t*-test. In agreement with this, we did not detect any significant differences concerning the white-matter fascicles as well. These findings can be suggestive about the way the brain adapts in response to lesions with a potentially different nature.

Trying to understand which factors might be responsible for the observed differences between iLGGs and sLGGs, we can exclude, first of all, lesion volume; the lesion growing pattern and the potentially greater involvement of the white matter seemed not to be the key factors, either. Demographic variables have to be excluded, too, given that gender, age, and years of education (together with handedness) did not significantly differ between the two groups. Although beyond the role of the current investigation, we may hypothesize a potential role of the molecular diagnosis, even though it partially differed from that reported in previous studies on iLGGs [7,12,15]. The limited sample size did not allow further investigations about the molecular profile role.

Further, we may also hypothesize an effect regarding more specific lesion locations, which can be inevitably slightly different between the two groups. As already proposed [2], it could occur that the iLGG lesions develop from less crucial or more easily compensated brain areas. Usually, LGGs take several months or years before manifesting with evident symptoms, as hypothesized from their usually large size at diagnosis. If they manifest early, when the lesion is still small, this means that they probably occurred in a trickier area. We conclude by stating that, instead of inspecting what makes iLGGs peculiar, it could be more interesting to investigate the reasons why some LGGs become symptomatic at an early stage.

As a last note, the findings we observed here with regard to the iLGGs can seem in partial contradiction with those observed in our previous reports, where the comparison was made with the healthy controls. However, as we also observed in other (still unpublished) investigations, the comparison between or within groups of patients may be more appropriate to address specific aspects, especially when dealing with plasticity associated with growing lesions.

A main limitation of this study is represented by the relatively small number of patients, which prevented us from running more in-depth analyses. Nonetheless, this limitation results from an inherent condition, given that iLGGs represent a rare condition. This factor also prevented a more thorough investigation of the factors that might have supported the observed findings. Given that little is still known about the effect that incidental lesions have on the brain, we think that the current study can contribute to fill this lack of knowledge, even if in exploratory terms. Indeed, in order to achieve a suitable sample size, multicenter studies are recommended to replicate these findings. Comparative next-generation sequencing studies are strongly required to investigate the potential role of the molecular and/or genetic profile between the iLGG and sLGG groups [2,4]. Besides these investigations, it would be also recommended to explore the effects in relation to other brain networks/functions.

## 5. Conclusions

This study contributed to shed more light on the factors that characterize iLGGs or, put another way, it prompts further investigation about the factors that make a lesion symptomatic, downsizing the role of increasing volume. Concerning the clinical relevance, these findings suggest that it is crucial to better understand if and how given areas of the brain and related circuits can be compensated when invaded by a slow-growing lesion. Indeed, early and optimized surgery [40] is considered the gold standard to treat not only sLGGs, but iLGGs too, as it was observed that in most of the cases, it was total or gross total and did not cause permanent postoperative deficits [2,7,21]. On the other hand, in the case of sLGGs with early clinical manifestations, it is fundamental to better understand the plastic potential an invaded structure can develop following surgery and the role of additional factors having a potential impact on this, in order to maximize the onco-functional balance.

## Figures and Tables

**Table 1:** Demographic and clinical information at the group level.

	sLGG	iLGG	Between-Group Difference
Sex	7 F, 6 M	5 F, 8 M	*χ*^2^ = 0.62, *df* = 1, *p* = 0.43 (*n.s*.)
Age (years)	*M* = 43.46, *SD* = 12.54	*M* = 37.46, *SD* = 12.54	*U* = 61.50, *p* = 0.24 (*n.s*.)
Education (years)	*M* = 12.00, *SD* = 4.51	*M* = 13.38, *SD* = 2.53	*U* = 70.00, *p* = 0.43 (*n.s*.)
Handedness ^1^	Right-handed = 12 Left-handed = 1	Right-handed = 12 Left-handed = 1	-
Lesion volume	22.31 cm^3^ (*SD* = 8.57 cm^3^; range = 10–36 cm^3^; median = 12 cm^3^)	18.54 cm^3^ (*SD* = 10.71 cm^3^, range = 8–40 cm^3^; median = 18 cm^3^)	*U* = 57.00, *p* = 0.16 (*n.s*.)
Lesion growth pattern	Expansive for all patients but one (p9, see Table 2)	Expansive for all patients but one (p3 [24])	-
Symptoms	Diverse symptoms and, particularly, epileptic seizures, which characterized all the patients in this group. 7 had language-related manifestations.	none	-
Object-naming abilities (BADA test [26])	score ≥ 28/30	score ≥ 28/30	-

Note. Between-group differences in demographic variables (i.e., Mann–Whitney U test and Pearson chi-square *χ*^2^ test) were computed on IBM SPSS Statistics, version 21 (www.ibm.com/SPSS) ^1^ and assessed by the Edinburgh Inventory [25]. The two left-handed patients had a naming network distribution comparable to that of the right-handed patients (i.e., they did not display right-hemisphere dominance).

**Table 3:** Results of the exclusive masking on iLGG and sLGG functional networks.

Cluster	Cluster Size (Voxels)	*T*	Macroanatomical Area	Cytoarchitectonic Localization	MNI Coordinates		
					x	y	z
iLGG masked exclusively by sLGG							
1.	188						
		4.73	L precentral gyrus	Area 44	−48	8	34
		5.14	L middle frontal gyrus	-	−50	24	32
		4.13	L inferior frontal gyrus	-	−46	26	24
2.	149						
		6.83	L superior temporal gyrus	Area PFcm (IPL)	−54	−38	22
		4.95	L supramarginal gyrus	Area PFcm (IPL)	−56	−36	26
3.	150						
		6.23	R inferior frontal gyrus	Area 45	52	28	20
		5.43	R middle frontal gyrus	-	52	26	32
sLGG masked exclusively by iLGG							
No suprathresholded clusters							

Note. Reported results were corrected for multiple comparisons at the cluster level (i.e., FWE, *p* < 0.05; height threshold of *p* < 0.001, uncorrected, at the voxel level).

**Table 4:** Results of the iLGG functional network masked inclusively by the sLGG network.

Cluster	Cluster Size (Voxels)	*T*	Macroanatomical Area	Cytoarchitectonic Localization	MNI Coordinates		
					x	y	z
iLGG masked inclusively by sLGG							
1.	7.975						
		12.85	R cuneus	Area hOc2 [V2]	15	−98	10
		11.74	L superior occipital gyrus	Area hOc3d [V3]	−16	−96	10
		9.73	L middle occipital gyrus	Area hOc4lp	−30	−86	4
		9.59	L fusiform gyrus	-	−36	−58	−10
		9.58	R middle occipital gyrus	Area hOc4lp	38	−84	10
		9.56	L middle occipital gyrus	Area hOc4d [V3A]	−24	−88	14
		9.31	R superior occipital gyrus	Area hOc3d [V3d]	20	−94	20
		8.94	R fusiform gyrus	Area FG3	30	−60	−6
2.	830						
		7.62	L precentral gyrus	-	−48	−4	42
		6.19	L precentral gyrus	Area 44	−48	8	32
		4.98	L inferior frontal gyrus	Area 45	−52	26	24
		4.81	L inferior frontal gyrus	Area 44	−52	12	26
3.	686						
		8.12	R posterior-medial frontal gyrus	-	2	14	56
		7.05	L posterior-medial frontal gyrus	-	−4	0	64
4.	646						
		7.77	R precentral gyrus	-	52	−2	48
		6.06	R inferior frontal gyrus	-	40	6	30
		5.87	R middle frontal gyrus	-	46	−2	54
5.	406						
		6.14	L thalamus	Thalamus: Visual	−24	−28	−4
		5.71	L hippocampus	-	−28	−28	−8
6.	317						
		6.60	R inferior frontal gyrus	Area 45	48	20	6
		6.39	R insula	-	32	28	4
		4.93	R inferior frontal gyrus	Area 44	52	14	2
7.	254						
		6.73	N/A	-	26	−32	8
		6.70	R hippocampus	-	24	−24	−10
8.	95	6.45	N/A	Thalamus: Temporal	24	−28	−2
		6.89	L inferior frontal gyrus	-	−34	32	0

Note. Reported results were corrected for multiple comparisons at the cluster level (i.e., N/A = not available; FWE, *p* < 0.05; height threshold of *p* < 0.001, uncorrected, at the voxel level).

**Table 5:** Results of the sLGG functional network masked inclusively by the iLGG network.

Cluster	Cluster Size (Voxels)	*T*	Macroanatomical Area	Cytoarchitectonic Localization	MNI Coordinates		
					x	y	z
sLGG masked inclusively by iLGG							
1.	8149						
		11.31	R cuneus	Area hOc2 [V2]	16	−98	12
		10.12	N/A	Area FG3	−35	−55	−8
		10.07	L middle occipital gyrus	Area hOc4d	−24	−88	14
		8.47	L middle occipital gyrus	Area hOc3d [V3]	−18	−96	10
		8.46	L inferior temporal gyrus	-	−44	−62	−6
		8.20	L inferior occipital gyrus	Area hOc4la	−46	−70	−4
		7.46	R angular gyrus	Area hIP3 (IPS)	28	−56	46
		7.36	R middle occipital gyrus	Area hOc4la	38	−80	8
2.	354						
		6.29	R precentral gyrus	-	44	8	32
		4.61	R inferior frontal gyrus	Area 45	54	20	25
		4.18	R middle frontal gyrus	-	44	−2	54
3.	310						
		5.88	L inferior frontal gyrus	-	−52	36	0
		5.64	L insula	-	−30	28	−2
		4.44	L inferior frontal gyrus	Area 44	−52	12	4
4.	283						
		6.65	N/A	-	26	−30	8
		6.24	L thalamus	Thalamus: Temporal	24	−28	−2
5.	265						
		5.70	L posterior-medial frontal gyrus	-	−4	0	64
		5.50	R posterior-medial frontal gyrus	-	8	12	48
6.	261		L inferior frontal gyrus	Area 44	−44	10	24
			L inferior frontal gyrus	Area 45	−54	28	16
7.	238						
		6.89	L thalamus	Thalamus: Temporal	−18	−32	2
8.	228	6.41	L thalamus	Thalamus: Visual	−26	−25	4
		6.25	R insula	-	34	20	6
		5.41	R inferior frontal gyrus	-	40	24	6
		4.31	R inferior frontal gyrus	Area 45	52	22	2
9.	222						
		5.68	L precentral gyrus	-	−50	0	46

Note. Reported results were corrected for multiple comparisons at the cluster level (i.e., N/A = not available; FWE, *p* < 0.05; height threshold of *p* < 0.001, uncorrected, at the voxel level).

**Table 6:** DTI parameters.

	ILF				IFOF				UF				Whole AF				Long AF			
	LH		RH		LH		RH		LH		RH		LH		RH		LH		RH	
	Num	FA	Num	FA	Num	FA	Num	FA	Num	FA	Num	FA	Num	FA	Num	FA	Num	FA	Num	FA
iLGG	277.37 (191.59)	0.47 (0.03)	244.72 (140.78)	0.45 (0.02)	173.55 (96.76)	0.49 (0.03)	308.36 (191.45)	0.49 (0.02)	227.55 (122.39)	0.42 (0.03)	238.28 (127.73)	0.42 (0.01)	555.63 (273.21)	0.45 (0.03)	598.82 (144.54)	0.46 (0.03)	198.18 (131.97)	0.47 (0.04)	132.36 (112.63)	0.46 (0.04)
sLGG	228.60 (134.45)	0.46 (0.04)	235.80 (125.25)	0.46 (0.03)	155.30 (117.18)	0.47 (0.05)	237.00 (88.39)	0.49 (0.03)	175.801 (47.64)	0.40 (0.03)	159.60 (81.86)	0.41 (0.03)	435.50 (252.15)	0.44 (0.04)	588.90 (208.55)	0.44 (0.03)	163.50 (137.90)	0.47 (0.03)	115.20 (103.53)	0.45 (0.03)

Note. Reported mean values (and SD, in parentheses) of numbers of streamlines and FA (fractional anisotropy) values for each fascicle in the left (LH) and right (RH) hemispheres. AF: arcuate fasciculus; IFOF: inferior fronto-occipital fasciculus; ILF: inferior longitudinal fasciculus; UF: uncinate fasciculus.

## Data Availability

Due to privacy reasons, data will be available upon request to the corresponding author.

## Supplementary Materials

The following supporting information can be downloaded at: https://www.mdpi.com/article/10.3390/brainsci14090887/s1. Table S1: Patients’ performance in the administered cognitive tasks; Table S2: Functional naming network in patients with sLGG; Figure S1: Object-naming-related functional activations [41,42,43,44].
