# Supplementary material for: Comparative Analysis of Brain Coping Mechanisms in Small Left-Hemisphere Lesions: Incidental vs. Symptomatic Gliomas
Source: Brain Sci. 2024 Aug 30;14(9):887. doi: 10.3390/brainsci14090887 (PMC11429952; doi:10.3390/brainsci14090887)
Supplement: Supplementary file 1 [file brainsci-14-00887-s001.zip › brainsci-3170280-supplementary.pdf]

Supplementary Table S1. Patients' performance in the administered cognitive tasks.

Patient	Verb naming	Word reading	Pseudo Word reading	Word repetition	Pseudo Word repetition	Word writing	Pseudo Word writing	Phonological discrimination	Verbal fluency	Verbal comprehension	Non-verbal intelligence	Verbal short-term memory	Verbal working memory
**1**	27	n/a	n/a	n/a	n/a	n/a	n/a	n/a	47	35	33	5.65	4.77
**2**	28	n/a	n/a	n/a	n/a	n/a	n/a	n/a	n/a	31.50	31	4.55	n/a
**3**	28	92	45	45	35	46	25	60	n/a	33.50	34	n/a	n/a
**4**	27	92	45	45	35	46	25	60	32	33	31	5.60	4.50
**5**	27	90	45	45	33	n/a	n/a	n/a	24	30.75	n/a	4.53	3.43
**6**	25	90	45	45	35	n/a	n/a	60	22	32.50	30	3.55	1.52
**7**	28	92	44	45	35	46	25	60	58	33.75	26	5.68	n/a
**8**	27	91	43	45	35	42	25	60	n/a	n/a	n/a	n/a	n/a
**9**	28	92	44	45	35	46	25	60	56	32.75	16	4.82	1.90
**10**	26	92	45	45	35	44	25	60	30	35	32.50	6.96	5.1
**11**	28	90	45	45	35	46	25	60	41	33.50	35.50	5.61	n/a
**12**	27	n/a	n/a	n/a	n/a	n/a	n/a	n/a	27	33.75	34	5.68	4.64
**13**	28	n/a	n/a	n/a	n/a	n/a	n/a	n/a	31	35	36	7	4
**Mean (SD) sLGGs**	27.23 (0.93)	91.22 (0.97)	44.57 (0.73)	45.00 (00.00)	34.68 (0.67)	45.14 (1.47)	25.00 (0.00)	60.00 (0.00)	36.80 (13.00)	33.33 (1.35)	30.82 (5.66)	5.48 (1.34)	3.73 (1.35)
**Mean (SD) iLGGs**	27.08 (1.66)	92.00 (00.00)	45.00 (00.00)	45.00 (00.00)	35.00 (00)	45.86 (0.38)	25.00 (0.00)	60.00 (0.00)	36.00 (12.43)	33.02 (1.24)	31.30 (2.82)	5.48 (1.03)	3.70 (0.73)
**t-test between iLGG and sLGG**	t = -.29; p = .77	t = 1.94; p = .07	t = 1.84; p = .10	-	t = .88; p = .40	t = 1.17; p = .28	-	-	t = -.15; p = .88	t = .26; p = .80	t = .26; p = .81	t = .11; p = .91	t = -.07; p = .95

Note.
n/a: not-available data.

Verb naming, word and pseudo-word reading, word and pseudo-word repetition, word and pseudo-word writing, and phonological discrimination were tests from the BADA battery [1]; for verbal fluency, we used a phonological fluency task [2]; for verbal comprehension, the Token test [3]; for non-verbal intelligence, the Raven matrices [4]; for verbal memory, digit span forwards and backwards, for short-term memory and working memory, respectively [5].
For verbal fluency and verbal comprehension, we computed the equivalent scores (i.e., an equivalent score of 0 means a pathological performance), after having corrected for age, gender, and education according to the published norms. We then calculated the corresponding equivalent scores to determine a pathological performance. For all the other tests for which normed conversion of raw into equivalent scores was not available, we compared them with the published cutoff values. Below-cutoff performance is highlighted in light gray. Individual data for patients with iLGG can be found in [6].

Supplementary Table S2. Functional naming network in patients with sLGG.

Cluster	Cluster size (voxels)	*T*	Macroanatomic area	Cytoarchitectonic localization	MNI coordinates		
					x	y	z
1.	8,461						
		10.67	R cuneus	Area hOc1 [V1]	16	-94	8
		9.93	L superior occipital gyrus	Area hOc3d [V3d]	-16	-94	8
		9.22	L middle occipital gyrus	Area hOc4lp	-26	-90	14
		9.15	R middle occipital gyrus	Area hOc4lp	38	-84	10
		8.43	R superior occipital gyrus	Area hOc4d [V3A]	24	-90	16
		9.95	L inferior temporal gyrus	-	-46	-58	-8
		7.60	R fusiform gyrus	-	26	-48	-12
		7.20	R inferior temporal gyrus	-	46	-58	-8
2.	512						
		7.40	R inferior frontal gyrus	-	46	10	30
		4.91	R inferior frontal gyrus	Area 45	56	18	28
		4.68	R precentral gyrus	-	38	-4	46
3.	373						
		6.53	N/A	Thalamus: visual	-26	-26	-4
		4.95	L thalamus	Thalamus: temporal	-18	-32	0

		4.43	L thalamus	Thalamus: prefrontal	-12	-12	-2
4.	327						
		5.48	L posterior-medial frontal gyrus	-	-4	12	52
		4.98	R posterior-medial frontal gyrus	-	6	10	48
5.	316						
		5.29	L precentral gyrus	-	-46	-4	42
6.	249						
		6.30	L inferior frontal gyrus	-	-42	10	26
		4.41	L inferior frontal gyrus	Area 45	-54	26	18
7.	241						
		6.67	N/A	Thalamus: parietal	22	-26	-4
		3.88	R hippocampus	-	30	-3	-8
8.	202						
		6.34	R insula	-	36	22	4
		4.34	R inferior frontal gyrus	-	50	18	-8
		4.32	R inferior frontal gyrus	Area 44	58	12	6

		3.90	R inferior frontal gyrus	Area 45	56	26	6
9.	164						
		4.66	L insula	-	-32	26	-4
		4.41	L inferior frontal gyrus	Area 44	-52	18	0
		3.86	L temporal pole	-	-48	18	-14

Note. Reported results were corrected for multiple comparisons at the cluster level (i.e., FWE, $p < .05$; height threshold of $p < .001$, uncorrected, at the voxel level).
N/A = not assigned.
Data for patients with iLGG can be found in [7].

Supplementary Figure S1. Object-naming-related functional activations.

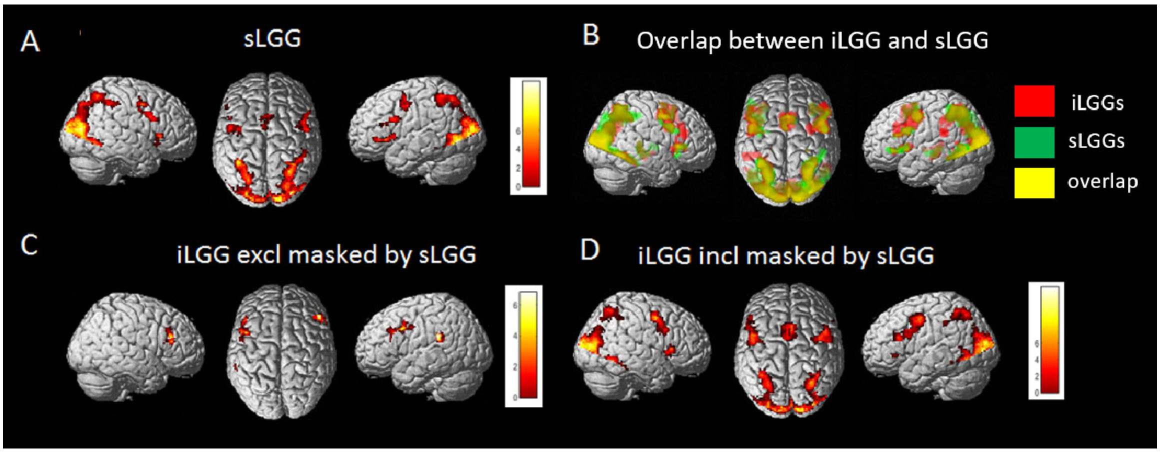

In A), rendered object- naming network of the sLGG group (for the iLGG group, see [7]; in B), rendered overlap between the object- naming networks of the two groups; in C) and D), rendered functional clusters resulting from exclusive and inclusive masking, respectively (in both cases, of iLGGs masked by sLGGs)

Note. Color bars indicate signal intensity.
